# Detection of Hidden Pest Rice Weevil (*Sitophilus oryzae*) in Wheat Kernels Using Hyperspectral Imaging

**DOI:** 10.3390/foods15030566

**Published:** 2026-02-05

**Authors:** Lei Yan, Taoying Luo, Chao Zhao, Honglin Ma, Yufei Wu, Chunqi Bai, Zibo Zhu

**Affiliations:** 1 School of Food and Strategic Reserves, Henan University of Technology, Zhengzhou 450001, China; luotaoying06@163.com (T.L.); 2018050@haut.edu.cn (C.Z.); wyf1135004574@163.com (Y.W.); baichunqi216@163.com (C.B.); 2 Henan Engineering Research Center of Intelligent Grain Storage, Zhengzhou 450001, China; 3 National Engineering Research Center of Grain Storage and Logistics, Zhengzhou 450001, China; 4 College of Information Science and Engineering, Henan University of Technology, Zhengzhou 450001, China2024920332@stu.haut.edu.cn (Z.Z.)

**Keywords:** hyperspectral imaging, stored wheat, *Sitophilus oryzae* infestation, feature selection, early detection, classification models

## Abstract

The rice weevil (*Sitophilus oryzae*) is a major pest in stored wheat, and traditional detection methods face challenges in identifying its hidden life stages within kernels. This study develops a nondestructive method to detect *S. oryzae* (*Sitophilus oryzae*) infestation in wheat kernels using hyperspectral imaging, spectral preprocessing, feature extraction, and classification modeling. Hyperspectral data were collected from wheat kernels at different infestation stages (1, 11, 21, and 25 days (d)) and from healthy kernels. Spectral quality was optimized using SG smoothing, multiplicative scatter correction (MSC), and standard normal variate transformation (SNV). Feature extraction algorithms, including Competitive Adaptive Re-weighting Algorithm (CARS), Successive Projection Algorithm (SPA), and Iterative Retention of Information Variables (IRIV), were used to reduce data dimensionality, while classification models like Decision Tree (DT), K-nearest neighbors (KNN), and Support Vector Machine (SVM) were applied. The results show that MSC preprocessing provides the best performance among the models. After feature band selection, the MSC-CARS-SVM model achieved the highest accuracy for the 1 day and 25 d samples (95.48% and 96.61%, respectively). For the 11 d and 21 d samples, the MSC-IRIV-SPA-SVM model achieved the best performance with accuracies of 94.35% and 94.92%, respectively. This study demonstrates that MSC effectively reduces spectral noise and improves classification performance. After feature selection, the model shows significant improvements in both accuracy and stability. The study confirms the feasibility of using hyperspectral technology to identify healthy and *S. oryzae*-infested wheat kernels, providing theoretical support for early, nondestructive pest detection.

## 1. Introduction

Postharvest loss during storage remains a critical bottleneck in safeguarding food security, with insect pests recognized as a principal driver of quantitative and qualitative grain deterioration. According to a survey by the Food and Agriculture Organization of the United Nations (FAO), approximately 10% of the global grain supply is infested by pests annually, with cryptic pests that feed within grain kernels being the most prevalent [1]. Among these, *S. oryzae* (*Sitophilus oryzae*) is the most common. It typically lays eggs inside the grain, where larvae develop undetected until adulthood, making early-stage detection highly challenging. In addition to quantitative losses, the excreta and residues left by *S. oryzae* also deteriorate grain quality [2]. Therefore, early detection of hidden grain storage pests, particularly before storage or during the early stages of infestation, is essential to minimizing postharvest losses.

Currently, the detection of hidden pests in stored grain is primarily guided by international standards ISO 6639-1:1986 and ISO 6639-4:1987 [3,4], which recommend several methods, including the baseline method, X-ray method, carbon dioxide method, anthrone colorimetric method, flotation method, and acoustic measurement method. While these techniques provide a degree of reliability and practical utility, they suffer from notable limitations such as lengthy detection times, low sensitivity, or demanding operational requirements [5,6]. With the advancement and integration of spectroscopy, biotechnology, and information technology, newer methods—such as enzyme-linked immunosorbent assay (ELISA) [7], biophoton detection [8], terahertz time-domain spectroscopy [9], and low-field nuclear magnetic resonance (LF-NMR)—have emerged. In particular, LF-NMR can exploit differences in proton relaxation characteristics between kernels and internal insects to detect and, in some cases, stage hidden *S. oryzae* infestation in wheat [10]. Terahertz (THz) imaging has also been used to monitor *S. oryzae*-infested grains by probing internal structural differences in relatively dry biological materials, and rapid screening may be achievable when combined with machine learning approaches [11]. However, despite their improved sensitivity and methodological diversity, these emerging techniques can still entail relatively complex workflows and/or higher barriers to routine on-site or online deployment (e.g., instrument cost, throughput limitations, and system integration requirements) [12,13]. By comparison, near-infrared hyperspectral imaging (NIR-HSI) is generally considered more amenable to conveyor-based inspection, offering chemically informative spectral–spatial data at a high acquisition speed and demonstrating feasibility for discriminating insect-damaged wheat kernels from sound kernels in similar NIR wavelength ranges [14].

Hyperspectral imaging (HSI) integrates the advantages of traditional imaging and spectroscopy, enabling the simultaneous acquisition of spatial and spectral information at high resolution for nondestructive detection. Due to its comprehensive data output and adaptability, HSI has been widely applied in agriculture in recent years, particularly for detecting crop diseases, pest infestations, and assessing seed quality [15]. Studies have demonstrated the ability of HSI to distinguish between healthy and pest-infested crops. For instance, Huang [16] used HSI combined with a Support Vector Machine (SVM) model to classify pest-infected soybeans with an accuracy of 97.3%, while Wang [17] successfully applied HSI to detect diseased maize seeds. A few studies have explored the use of HSI for detecting stored grain pests. Zhang constructed a near-infrared hyperspectral imaging system to extract characteristic wavelengths for identifying grain borers [18], and Cao combined HSI with a back-propagation neural network to distinguish between maize weevils and *S. oryzae* [19]. However, most of these studies focus on adult insects or externally visible stages, with limited attention to the internal, immature stages of infestation. Furthermore, existing studies often rely on spectral data from a single time point, lacking a dynamic understanding of pest developmental stages. These limitations hinder the broader application of HSI in the early detection of cryptic storage pests.

In this study, healthy wheat kernels and wheat kernels infested with *S. oryzae* at different developmental stages were investigated. Hyperspectral images were acquired, and mean spectra were obtained by averaging the reflectance of all pixels within each kernel region. By integrating multiple spectral preprocessing techniques, feature band selection methods, and classification algorithms, a nondestructive identification model was developed to detect hidden *S. oryzae* infestation in wheat kernels. The novelty of this work lies in proposing and validating a hyperspectral imaging-based nondestructive workflow that enables multi-time point identification of hidden *S. oryzae* infestation at the single-kernel level, and in systematically comparing preprocessing and feature selection strategies to determine robust and efficient modeling combinations. This work aims to provide a theoretical basis and methodological reference for applying hyperspectral imaging to the early, nondestructive detection of hidden insect infestations during grain storage.

## 2. Materials and Methods

### 2.1. Experimental Materials

Zhengmai 119 was purchased from Yanjin County in 2017 (Henan, China). Before the experiment, the wheat was stored at 4 °C in a refrigerator. To remove residual insecticides and eliminate any potential insect eggs, the wheat grains were washed with water three times and then dried in a forced-air drying oven (Beijing Yongguangming Medical Instrument Co., Ltd., Beijing, China) at 65 °C for 2 h. After cooling to room temperature, the moisture content was adjusted to 13.0 ± 0.5%, and the grains were sealed in self-sealing bags for subsequent use.

### 2.2. Hyperspectral Imaging Systems

**Figure 1:**
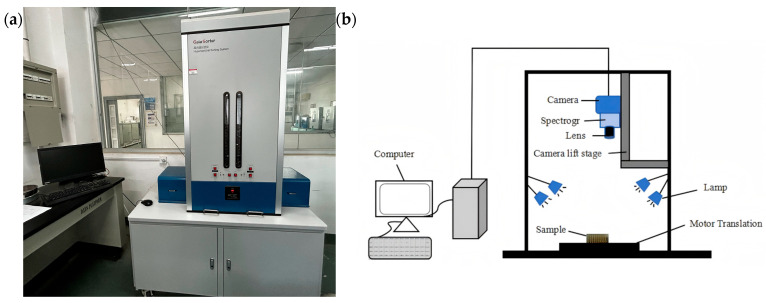
Hyperspectral imaging system. (**a**) Photograph; (**b**) schematic.

A Gaia Sorter-Dua hyperspectral imager (Shuangli Herspectrum Technology Co., Ltd., Chengdu, China) was used in this study. The physical layout and schematic diagram of the system are shown in Figure 1. The system operates in a push-broom imaging mode, with a spectral acquisition range of approximately 850–1700 nm. It is equipped with a camera resolution of 320 × 256 pixels, a spectral resolution of 5 nm, and includes 256 spectral channels. The main components of the system include a CCD camera, spectrometer, lens, halogen light source, electronically controlled translation stage, and a computer system. 

### 2.3. Sample Preparation

Two hundred grams of intact wheat kernels with full grains, uniform size, and no visible damage or wormholes was selected and exposed to 600 adult *S. oryzae* of the same developmental stage, approximately two weeks post-emergence. To obtain wheat samples infested with *S. oryzae* eggs, the adults were allowed to oviposit for 48 h in a dark incubator maintained at 29 ± 1 °C and 74 ± 2% relative humidity, thereby promoting synchronized subsequent development and enabling clearer comparisons among sampling time points. After the incubation period, all adult weevils were removed. Simultaneously, uninfected wheat kernels were prepared as a control group for the experiment.

### 2.4. Hyperspectral Image Acquisition and Spectral Extraction

**Figure 2:**
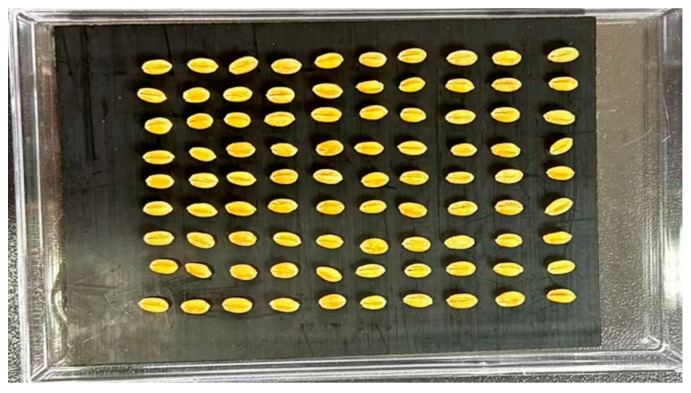
Sample placement.

Two d after the infestation treatment, all adult *S. oryzae* were removed. A total of 1220 wheat kernels—including 1000 mixed (potentially infested and uninfested) grains and 220 confirmed healthy grains—were selected and placed onto the mounting plate. Due to the natural groove on the ventral groove side of the wheat kernel, which provides a protective site preferred by *S. oryzae* for oviposition, all kernels were positioned with the ventral groove facing upward. Hyperspectral images were acquired from this orientation, as illustrated in Figure 2. The samples were incubated under dark conditions at a temperature of 29 ± 1 °C and relative humidity of 74 ± 2%. The first hyperspectral scan was conducted immediately after adult removal and recorded as day 1. Subsequent imaging was performed at two-day intervals to ensure that both healthy and potentially infested kernels were scanned simultaneously, thereby minimizing the impact of moisture variation on spectral data. This process continued until adult *S. oryzae* emerged from some of the kernels. The locations of these emergent adults were recorded, and the corresponding kernels were identified as infested. Ultimately, 369 wheat kernels were confirmed to be infested based on adult emergence and were used alongside the 220 healthy kernels for further analysis. According to the developmental timeline of *S. oryzae*, the egg stage lasts for approximately 9 d, the larval stage spans d 10 to 20, the pupal stage from d 21 to 26, and the adult stage from d 27 to 30 [20]. Therefore, in this study, spectral data collected on d 1, 11, 21, and 25 were selected for analysis, corresponding to the egg, larval, pupal, and emerging adult stages, respectively.

To eliminate the effects of uneven light source distribution, camera dark current, and external noise during wheat hyperspectral image acquisition, black-and-white correction was performed on the acquired hyperspectral images after each sample scan [21]. The correction was applied using the formula shown in Equation (1):

(1) $$I=\frac{I_{0}-B}{W-B}$$

where *I* denotes the corrected hyperspectral image, *I*_0_ represents the original (uncorrected) sample image, *B* is the dark reference (blackboard) image, and *W* is the white reference (whiteboard) image.

**Figure 3:**
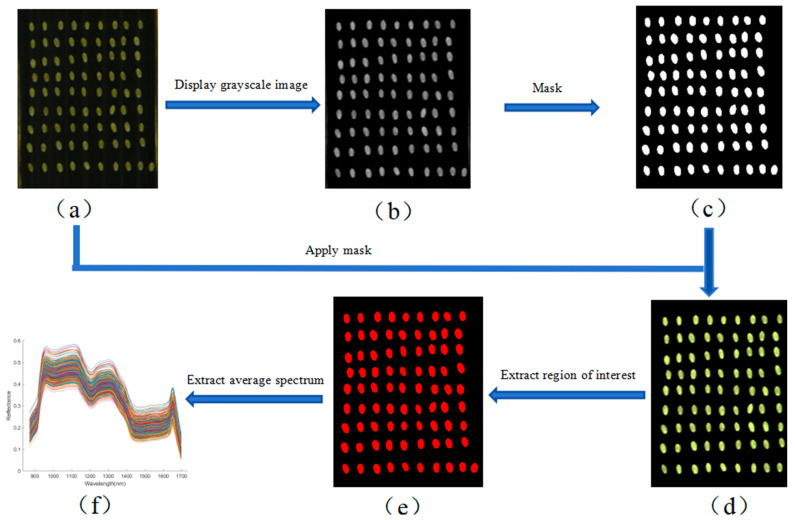
Spectral extraction workflow for wheat kernels. (**a**) Cropped hyperspectral image; (**b**) gray scale; (**c**) binary; (**d**) mask; (**e**) region of interest; (**f**) mean spectrum. Different colors indicate different samples (same below).

After obtaining the calibrated hyperspectral images, background removal and spectral data extraction of the wheat kernels were performed. This process included image resizing, mask generation and application, and region of interest (ROI) extraction. Image resizing and mask generation were carried out using ENVI 5.6 software, while ROI feature extraction was performed in MATLAB R2023a. According to Equation (2), the average spectral reflectance of all pixels within each ROI was calculated and used as the representative spectrum of the corresponding wheat kernel [22]. The complete extraction workflow is illustrated in Figure 3. As shown in Figure 3f, the average spectral curve was derived based on Equation (2), with wavelength on the horizontal axis and reflectance on the vertical axis.

(2) $$P=\frac{\sum_{k}^{n}\sum_{t}^{m}W_{kt}}{n}$$

where *P* denotes the average spectral value of the region of interest (ROI); *n* is the total number of pixels within the ROI; *m* represents the total number of spectral bands; and *W_kt_* is the spectral value of the *k*-th pixel at the *t*-th spectral band.

### 2.5. Data Processing

#### 2.5.1. Spectral Preprocessing

Raw spectral data often contain background signals or noise originating from the instrument, light source, or surrounding environment. These interferences can result in spectral distortion and baseline drift. Spectral preprocessing is essential to mitigate such adverse effects, enabling smoother signals, more stable baselines, and the reduction in multiple scattering effects in the spectral data [23]. In this study, seven preprocessing algorithms were employed to enhance spectral quality: Savitzky–Golay smoothing (SG) [24], multiplicative scatter correction (MSC) [25], standard normal variate (SNV) [26], first-order Savitzky–Golay derivative (SG-FD), second-order Savitzky–Golay derivative (SG-SD) [27], detrending method (Det), and baseline correction (BC) [28].

#### 2.5.2. Band Screening

The hyperspectral images acquired in this study consist of 256 spectral bands. When using the full spectrum for modeling, the analysis can be time-consuming, and the high correlation among bands may lead to information redundancy. Therefore, it is essential to perform dimensionality reduction or feature selection on the original spectral data to improve modeling efficiency and accuracy [29]. To this end, several feature selection algorithms were employed, including Competitive Adaptive Reweighted Sampling (CARS) [30], Successive Projections Algorithm (SPA) [31,32], Uninformative Variable Elimination (UVE) [33], and Iterative Retained Informative Variables (IRIV) [34,35]. In addition, combined feature selection approaches, such as CARS-SPA, UVE-SPA, and IRIV-SPA, were also used to identify informative wavelengths for spectral data analysis.

These four variable selection algorithms were chosen because they offer complementary advantages for handling high-dimensional and highly collinear hyperspectral data. CARS, driven by PLS regression coefficients and a “rapid elimination–refinement” strategy, is effective for removing large numbers of uninformative or noisy wavelengths at an early stage. UVE and IRIV focus on the stability and information content of variables under repeated resampling, and are therefore suitable for fine-grained screening of truly informative wavelengths. SPA uses a forward projection strategy to minimize multicollinearity among the selected bands, and can be applied as a second-stage optimization tool. In this study, SPA was used both alone and in combination with CARS, UVE, and IRIV (CARS–SPA, UVE–SPA, and IRIV–SPA) to obtain compact yet informative wavelength subsets, which are beneficial for subsequent model generalization and potential implementation in real-time or online detection systems.

#### 2.5.3. Model Building and Evaluation

To identify wheat samples infested with *S. oryzae* eggs, this study developed qualitative classification models to determine infestation status. Common discriminative approaches for qualitative classification include Logistic Regression [36], Linear Discriminant Analysis (LDA) [37], Support Vector Machines (SVMs), K-nearest neighbors (KNN), Random Forests (RFs) [38,39,40], and Artificial Neural Networks (ANNs). Linear models are generally suitable for problems with simple or approximately linearly separable feature distributions, whereas nonlinear models are better suited to complex, high-dimensional, and nonlinear data. Because egg infestation may alter kernel moisture, protein composition, and internal structure, thereby inducing nonlinear variations in spectral responses, four widely used nonlinear classifiers—SVM, Decision Tree (DT) [41], KNN, and RF—were selected for infestation identification. Model performance was evaluated using classification accuracy (training and test sets) and the Kappa coefficient, where higher values indicate better performance. SVM performs classification by constructing an optimal separating hyperplane. In this study, an SVM with a radial basis function (RBF) kernel was implemented, with the penalty parameter (Cost) set to 2.5 and the kernel scale (KernelScale) set to “auto”. To determine the optimal SVM hyperparameters, a grid search combined with 5-fold cross-validation was conducted on the training set. A DT classifier was employed with the Gini index as the splitting criterion and a maximum of 20 splits. KNN assigns labels by majority voting among the K-nearest neighbors; here, Minkowski distance with equal neighbor weighting was used. The optimal K was selected from {3, 5, 7, 9, 11, 15, 20} using 5-fold cross-validation on the training set by jointly considering accuracy and Kappa, thereby improving the transparency and reproducibility of K selection. Finally, the RF model was built using 200 decision trees with a minimum leaf size of 5 to enhance robustness and reduce overfitting [42].

To evaluate model performance, three key metrics were used as follows: accuracy (*Acc*), F1-score, and the Kappa coefficient, assessed on both the training and testing datasets. Higher accuracy values, ideally approaching 100%, indicate better classification performance. Similarly, F1-scores and Kappa values closer to 1 suggest stronger consistency between predicted and actual results. The formulas for these evaluation metrics are presented as follows.

(3) $$\mathrm{Acc}/\%\;=\frac{TP+\mathrm{TN}}{TP+\mathrm{TN}+\mathrm{FP}+\mathrm{FN}}\;\times \;100$$

(4) $$\mathrm{Precision}=\frac{TP}{\mathrm{TP}+\mathrm{FP}}$$

(5) $$R\mathrm{ecall}=\frac{\mathrm{TP}}{TP+FN}$$

(6) $$F1\;\text{-}\;\mathrm{score}=2\;\times \;\frac{\mathrm{Precision}\;\times \;Recall}{\mathrm{Precision}+\mathrm{Recall}}$$

*TP* is the number of true positives, i.e., the number of genuine samples that have been correctly identified; *FN* is the number of false negatives, i.e., the number of genuine samples that have been incorrectly identified as false; *FP* is the number of false positives, i.e., the number of false samples that have been incorrectly identified as true; and *TN* is the number of true negatives, i.e., the number of false samples that have been correctly identified as false.

## 3. Results

### 3.1. Average Spectra of Samples from Different Time Periods

**Figure 4:**
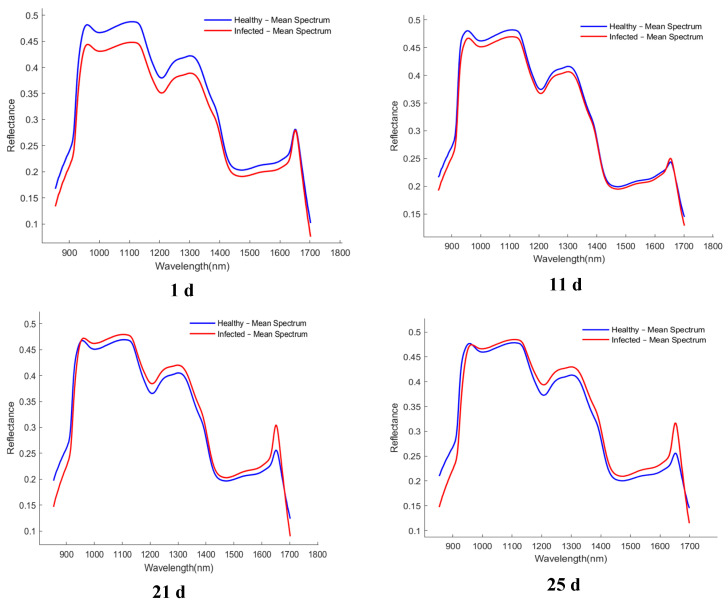
Average spectrum of healthy and infected samples collected at different time periods. The *x*-axis denotes wavelength (nm), and the *y*-axis denotes reflectance (dimensionless).

The average spectral curves of healthy and infected wheat samples collected at different time points (1, 11, 21, and 25 d) are presented in Figure 4. Although the overall spectral trends of the healthy samples (blue line) and the infected samples (red line) were similar, and noticeable and consistent differences in reflectance were observed in specific wavelength regions. These differences were primarily concentrated in the spectral ranges of 980–1100 nm, 1200–1300 nm, and 1400–1600 nm.

### 3.2. Selection of Preprocessing Methods

**Figure 5:**
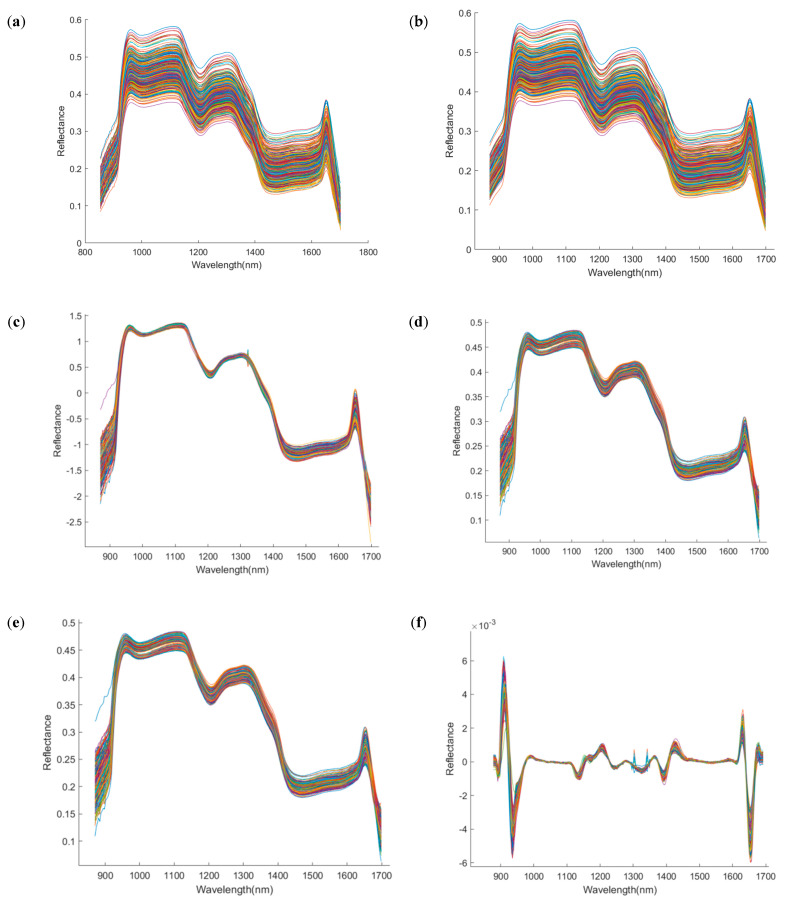

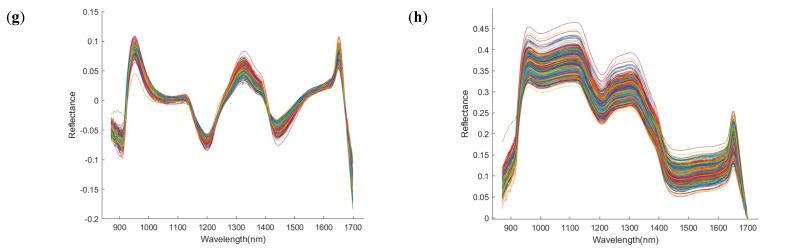
Spectral images after preprocessing by different methods. The *x*−axis denotes wavelength (nm), and the *y*−axis denotes reflectance (dimensionless). (**a**) Raw; (**b**) Savitzky–Golay; (**c**) standard normal variate; (**d**) multiplicative scatter correction; (**e**) Savitzky−Golay first derivative; (**f**) Savitzky−Golay second derivative; (**g**) detrend; (**h**) baseline correction.

To reduce the interference of non-quality information in the spectral data, various preprocessing techniques were applied to the raw spectra, with the results shown in Figure 5. Compared with the raw spectra (RAW), Savitzky–Golay smoothing (SG) produced smoother spectral curves; MSC and SNV effectively reduced scattering effects and improved curve stability; derivative-based methods (e.g., SG-FD, SG-SD) enhanced peak features but also amplified noise; while Det and BC improved the consistency across spectral profiles. Subsequently, the Kennard–Stone (K-S) algorithm was used to divide the samples into training and test sets in a 7:3 ratio. To enhance model robustness, five-fold cross-validation was employed during the training phase. Based on the different preprocessing methods, four classification models were constructed, and their classification accuracies and Kappa coefficients were evaluated across four time points: day 1, day 11, day 21, and day 25. The results are summarized in Table 1, Table 2, Table 3 and Table 4.

As shown in Table 1, Table 2, Table 3 and Table 4, the SVM and RF models exhibited higher classification accuracy and Kappa values across all time points, significantly outperforming the DT and KNN models. This suggests that SVM and RF are better suited for capturing the potential nonlinear relationships within the spectral features of wheat kernels. Among these, the SVM model demonstrated the best performance under MSC preprocessing, achieving test set accuracies of 93.22%, 91.53%, 94.35%, and 94.92% on d 1, 11, 21, and 25, respectively, exceeding 90%. The corresponding Kappa coefficients were 0.8542, 0.8290, 0.8807, and 0.8916, consistently above 0.80. The results also indicate that the models are sensitive to the type of spectral preprocessing applied. Compared with raw spectra (RAW), appropriate preprocessing significantly improved model performance. In particular, SNV, MSC, and SG−FD were especially effective. For example, under the RF model at day 25, MSC and SNV preprocessing yielded accuracies of 93.22% and 90.35%, with Kappa coefficients of 0.8567 and 0.8504, respectively. In contrast, models using unprocessed (RAW) or only SG-smoothed spectra performed less effectively, especially the DT model, where test set accuracy generally remained below 90%. In addition, classification performance tended to improve with increasing sampling time, with models showing noticeably better results at d 21 and 25. These results indicated that SVM emerged as the best−performing classification model in this study, and MSC preprocessing consistently produced the highest test set accuracy across all models. Therefore, MSC was identified as the optimal preprocessing method for this application.

### 3.3. Extraction of Characteristic Wavelengths

#### 3.3.1. Competitive Adaptive Reweighting Method (CARS) to Extract Feature Wavelengths

**Figure 6:**
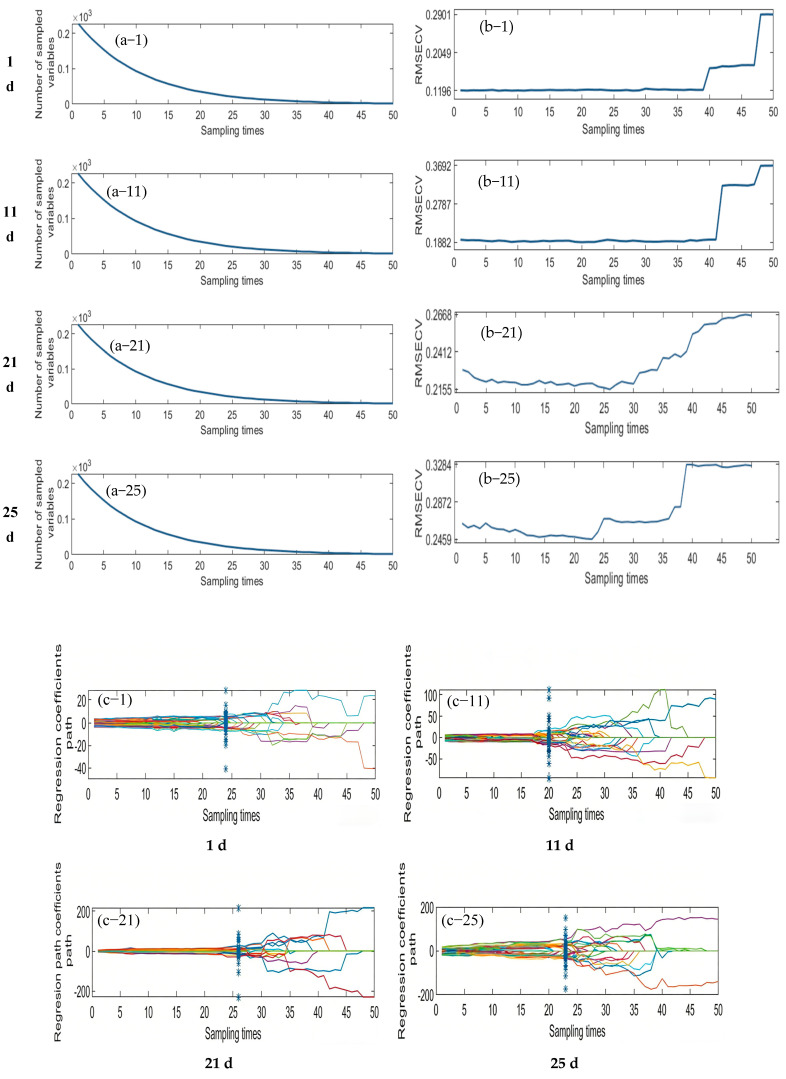
Process diagram of feature wavelength selection using the CARS algorithm. (**a**) Number of selected variables; (**b**) RMSECV curve; (**c**) regression coefficient trajectories. d 1, 11, 21, and 25 denote the sampling time points (Day 1, Day 11, Day 21, and Day 25).

To extract informative spectral bands using the CARS algorithm, multiple repeated experiments were conducted to determine the optimal parameter combination that minimized the root mean square error of cross−validation (RMSECV). The final parameters were set as follows: the maximum number of principal components was limited to 8, the number of Monte Carlo sampling iterations was 50, and a partial least squares (PLS) model was constructed using ten-fold cross−validation. Figure 6 illustrates the variation in the number of variables, the RMSECV curves, and the regression coefficient trajectories during the CARS−based feature selection process for the samples collected on d 1, 11, 21, and 25. As shown in Figure 6a, the number of selected variables decreases progressively with the number of sampling iterations, dropping rapidly in the initial stages and gradually stabilizing later. This trend reflects the core strategy of the CARS algorithm: “rapid elimination followed by fine optimization.” Figure 6b shows that the RMSECV curves generally follow a downward-then-upward pattern, indicating that the removal of redundant variables in the early stages improves model performance, while excessive elimination in the later stages may result in the loss of critical information and thus increase prediction error. Figure 6c presents the path diagrams of the regression coefficients, which provide insights into the distribution and stability of the selected key variable. Specifically, the optimal point for the day 1 sample occurred at the 24th iteration, with 58 characteristic bands selected. For the day 11 sample, the lowest RMSECV was achieved at the 20th iteration, with 38 bands retained. The day 21 sample reached its optimal performance at the 26th iteration, yielding 60 bands, and the day 25 sample performed best at the 23rd iteration, with 53 bands selected.

#### 3.3.2. Successive Projection Algorithm (SPA) to Extract Feature Wavelengths

**Figure 7:**
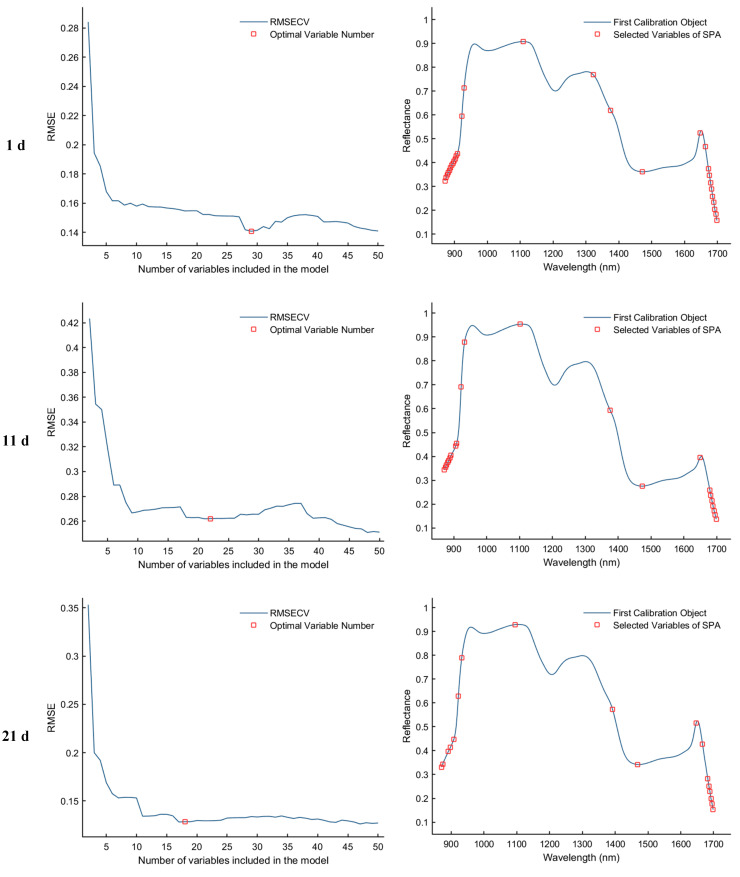

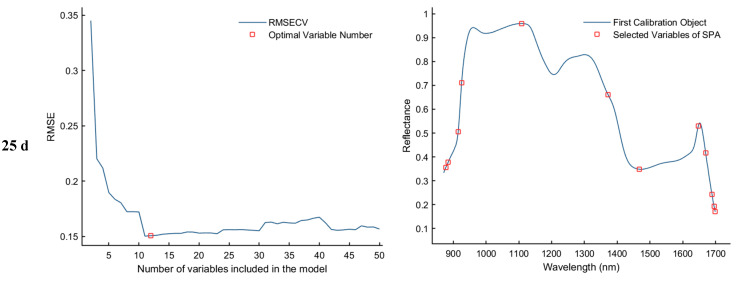
Feature wavelength selection results using the SPA algorithm.

The Successive Projections Algorithm (SPA) eliminates multicollinearity between spectral bands through a forward variable selection strategy. When the RMSECV reaches its minimum value, the selected feature bands exhibit the lowest redundancy and weakest inter-band correlation, making them more representative of the sample characteristics. As illustrated in Figure 7, the SPA−based feature selection process is presented for the samples collected on d 1, 11, 21, and 25. The first column of plots shows the RMSECV curves corresponding to different numbers of selected variables, which are used to determine the optimal number of features. The second column displays the distribution of the selected feature bands within the average spectra. Specifically, for the day 1 sample, the optimal model performance was achieved when the RMSECV reached 0.1407, resulting in the selection of 29 feature bands. For the day 11 sample, the minimum RMSECV was 0.2611 with 22 feature bands selected. In the day 21 sample, the lowest RMSECV value of 0.1285 corresponded to 18 selected bands. Finally, for the day 25 sample, the optimal performance was observed at an RMSECV of 0.1507, with 12 discriminative feature bands identified.

#### 3.3.3. Uninformative Variable Elimination Transform (UVE) Method for Extracting Feature Wavelengths

**Figure 8:**
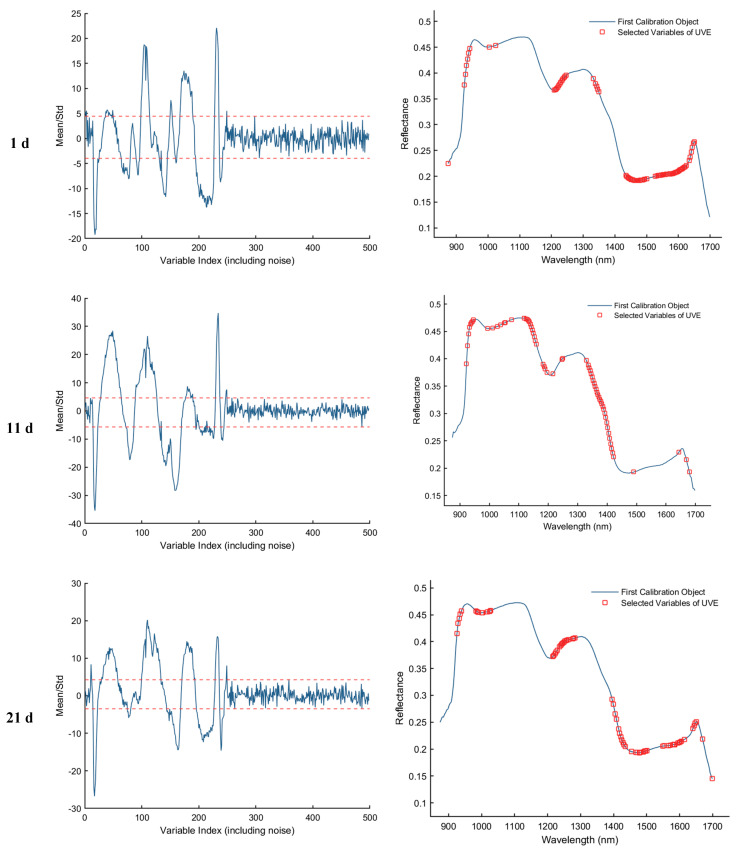

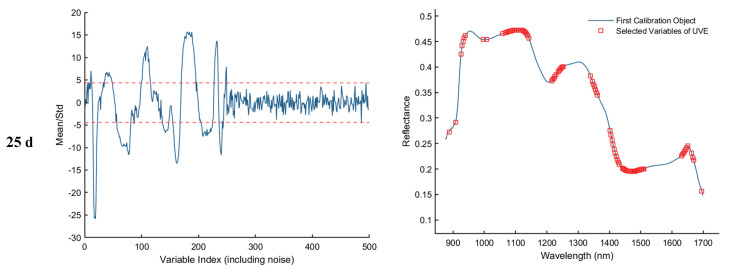
Feature wavelength selection results using the UVE algorithm. The red dashed lines indicate the upper and lower thresholds.

As shown in Figure 8, the results of feature wavelength selection using the UVE algorithm for the samples collected on d 1, 11, 21, and 25 are presented. The first column of plots illustrates the variable stability analysis, where the two horizontal dashed lines represent the upper and lower thresholds. Variables falling within these thresholds are considered uninformative or redundant and are eliminated, while those exceeding the thresholds are retained as informative feature wavelengths. The second column of plots displays the spectral distribution of the retained feature bands after UVE screening. As a result, a total of 82, 66, 68, and 94 informative wavelengths were selected for the 1st, 11th, 21st, and 25th day samples, respectively.

#### 3.3.4. Iterative Retention of Information Variables (IRIV) Method of Feature Wavelength Extraction

**Figure 9:**
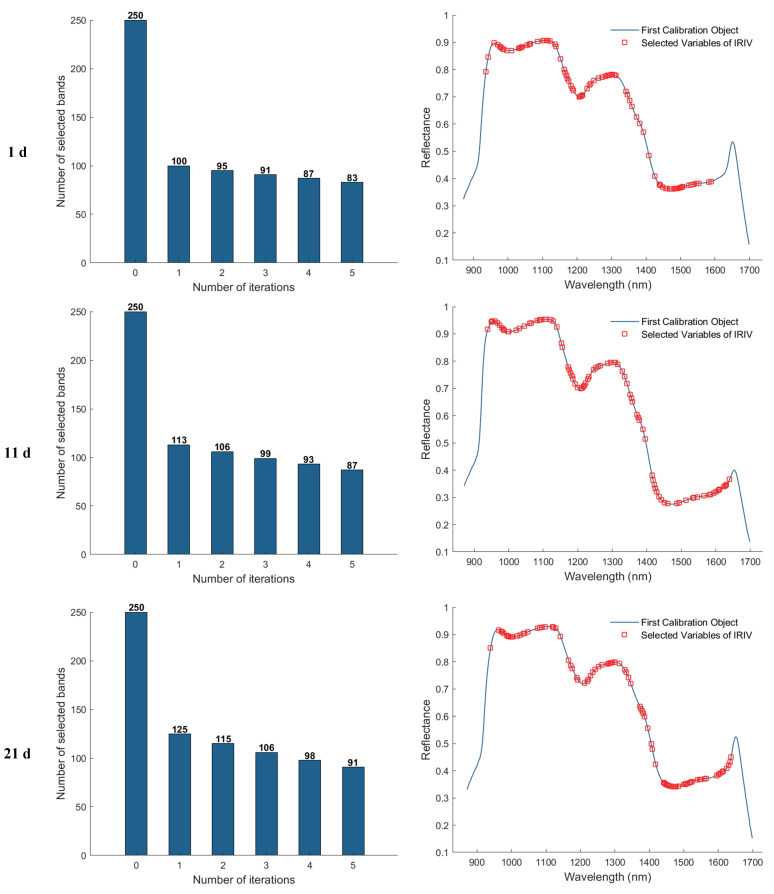

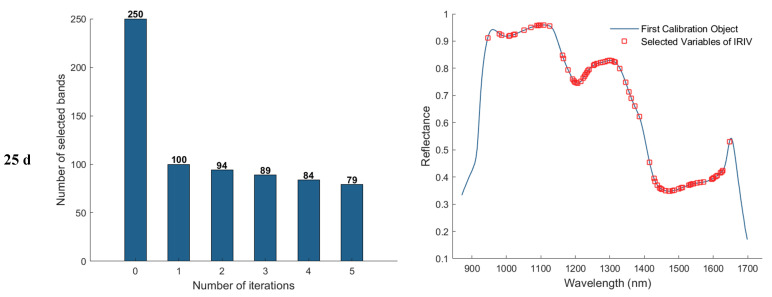
Feature wavelength selection results using the IRIV algorithm.

In the feature band selection process using the Iterative Retained Informative Variables (IRIV) algorithm, the maximum number of principal components was set to 40. A partial least squares (PLS) model was constructed using 11−fold cross-validation, with RMSECV employed as the evaluation metric for model performance. Figure 9 illustrates the variable selection process for the 1, 11, 21, and 25 d samples using IRIV, as well as the distribution of the selected feature bands in the average spectra. The first column of plots shows that the IRIV algorithm conducts multiple iterations, where each iteration builds a PLS model using the current subset of variables, evaluates its RMSECV, and updates the selection by retaining the optimal variable combination before proceeding to the next round. Through this iterative process and the inverse elimination of redundant variables, a stable and informative set of feature bands is ultimately obtained. The second column of plots displays the locations of the selected feature bands in the spectrum after IRIV screening. As a result, the number of selected eigen−wavelengths for the 1, 11, 21, and 25 d samples was 81, 92, 102, and 86, respectively.

#### 3.3.5. Joint Feature Wavelength Extraction Method

Although the number of spectral variables was significantly reduced by the CARS, UVE, and IRIV algorithms, the average number of selected variables remained relatively high−approximately 52, 76, and 90, respectively−across different periods. Such a high dimensionality is not conducive to the practical implementation of real-time or online detection devices. Therefore, in this study, the SPA was further applied to the feature bands initially selected by CARS, UVE, and IRIV to reduce both multicollinearity and the number of feature wavelengths. The results of the combined feature selection approach are presented (Table 5). Compared with the use of single feature selection methods, the joint method achieved a substantial reduction in the number of selected bands, thereby improving model simplicity and the feasibility of online deployment.

### 3.4. Comparison of Characteristic Wavelength Modeling

To compare the classification performance of different feature selection methods, seven approaches−including CARS, SPA, UVE, IRIV, and their respective combinations with SPA−were employed in this study. These were combined with four classification models: DT, KNN, SVM, and RF, to model the data collected at four sampling time points: d 1, 11, 21, and 25. Model performance was primarily evaluated using the classification accuracy and Kappa coefficient of the test set, as summarized in Table 6, Table 7, Table 8 and Table 9.

Among all the models, SVM demonstrated the best overall performance, with test set accuracies exceeding 90% at all time points and Kappa coefficients ranging from 0.85 to 1.00 (Table 8). Specifically, for the samples collected on d 1 and 25, the SVM model achieved the highest accuracy using feature bands selected by CARS. On d 11 and 21, the best results were obtained using feature bands selected by the IRIV-SPA method. The RF models also showed high classification performance, with the best results achieved using UVE-SPA on d 1, 11, and 21, and the UVE method alone on day 25 (Table 9). In contrast, the KNN and DT models exhibited relatively weaker performance. Their test set accuracies were generally lower than those of the SVM and RF models, with correspondingly lower Kappa coefficients. The DT model, in particular, consistently achieved accuracies below 91% and exhibited a large discrepancy between accuracy and Kappa coefficient values. For the KNN model, the best performance on day 1 was achieved using CARS−SPA, while the SPA method yielded the best results for the remaining time points (Table 7). In the DT model, UVE−SPA performed best on d 1 and 25, while CARS−SPA was optimal on d 11 and 21 (Table 6).

In summary, the comparative performance of the four classification models based on different feature selection methods ranked as follows: SVM > RF > KNN > DT. The SVM model consistently outperformed the others, both in full−spectrum analysis and when applied to selected feature bands. Notably, on d 1 and 25, CARS−based feature selection yielded the best SVM performance, with test set accuracies of 95.48% and 96.61%, and Kappa coefficients of 0.9050 and 0.9284, respectively. On d 11 and 21, IRIV−SPA-based feature selection achieved the best results, with test set accuracies of 94.35% and 94.92%, and Kappa coefficients of 0.8813 and 0.8922, respectively. Compared to full−spectrum models, the feature selection-based SVM models demonstrated improved accuracy and Kappa values. These findings indicate that appropriate feature selection methods can effectively reduce spectral covariance and redundancy, remove irrelevant information, and enhance the classification performance and robustness of the model.

### 3.5. Analysis of SVM Model Classification Performance Under Different Feature Inputs

**Figure 10:**
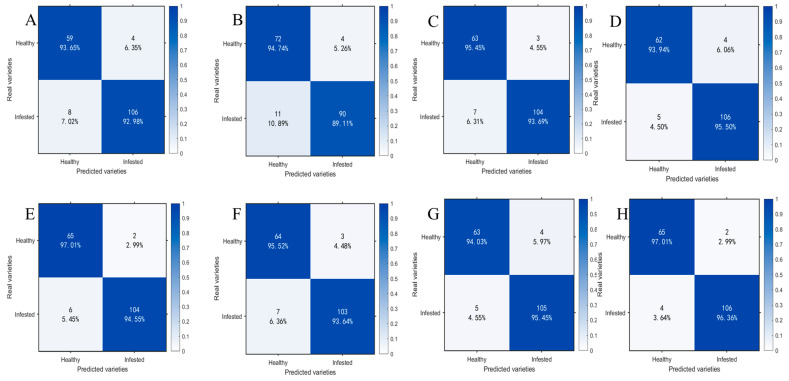
Test set confusion matrices for SVM models under different feature inputs. (**A**) 1 d−MSC; (**B**) 11 d−MSC; (**C**) 21 d−MSC; (**D**) 25 d−MSC; (**E**) 1 d−MSC−CARS; (**F**) 11 d−MSC−IRIV−SPA; (**G**) 21 d−MSC−IRIV−SPA; (**H**) 25 d−MSC−CARS.

To compare the classification performance of models before and after spectral preprocessing and after feature wavelength extraction, this paper presents the prediction results of the optimal SVM classification models obtained at each stage in the form of confusion matrices and conducts an accuracy analysis. Figure 10 presents a comparison of confusion matrices under different feature input conditions: A−D correspond to SVM models trained solely on MSC-preprocessed samples at 1 day, 11 d, 21 d, and 25 d, respectively; E and H represent SVM models trained on feature bands extracted from CARS; and F and G represent SVM models trained on feature bands extracted from IRIV-SPA. The horizontal axis denotes predicted classes, the vertical axis denotes true classes, diagonal elements indicate classification accuracy for each class, and off-diagonal elements reflect the distribution of misclassified samples.

By comparing the confusion matrices of SVM models that had been trained on preprocessed data with those of models built after additional feature band extraction, it was found that classification performance was significantly enhanced across all time points (1 d, 11 d, 21 d, and 25 d). The test set accuracy for the 1 d samples was increased from 93.22% to 95.48%, for 11 d from 91.53% to 94.35%, for 21 d from 94.35% to 94.92%, and for 25 d from 94.92% to 96.61%. The corresponding F1−scores were also improved. At the same time, the numbers of misclassified healthy and infected samples were reduced by 4, 5, 1, and 3 for the 1 d, 11 d, 21 d, and 25 d samples, respectively. These results indicated that, after feature band extraction was introduced, the model’s discriminative capability and stability in distinguishing samples with minor spectral differences were enhanced.

## 4. Discussion

In this study, hyperspectral imaging was integrated with spectral preprocessing and feature wavelength selection to enable nondestructive identification of hidden *S. oryzae* infestation in wheat kernels. The mean spectra of healthy and infested kernels showed consistent reflectance differences over several wavelength intervals. These differences may be related to infestation-driven changes in kernel internal structure and composition (e.g., moisture status and major constituents such as starch and proteins), which can jointly affect absorption and scattering in the NIR region [43,44]. The most apparent separations occurred in 980–1100, 1200–1300, and 1400−1600 nm, which are often considered chemically informative in the short-wave NIR. The 970−1000 nm region is commonly associated with water-related O–H absorption and may reflect moisture variation in cereal kernels [45]. The 1100–1300 nm interval is frequently attributed to C–H second overtones, whereas features in 1400–1600 nm are generally linked to O–H/N–H first overtones; thus, these regions may respond to both water status and major organic constituents [46]. Overall, the wavelength-dependent patterns observed here are consistent with prior studies indicating that NIR hyperspectral imaging can capture kernel-scale differences nondestructively and can support cereal quality assessment, including the discrimination of insect-damaged kernels in similar spectral ranges [47].

Preprocessing improved model performance to varying degrees compared with raw spectra, indicating that kernel hyperspectral signatures are influenced not only by chemical absorption but also by scattering and baseline variability. Such effects can arise from kernel heterogeneity, surface micro-roughness, subtle differences in illumination/collection geometry, and instrument noise. Under the present conditions, multiplicative scatter correction (MSC) provided the most consistent improvement in test set performance across models. This may be explained by its ability to reduce multiplicative and additive distortions, thereby enhancing absorption-related information that is more closely associated with kernel composition and improving inter-sample contrast [14]. Standard normal variate (SNV) also yielded favorable results, particularly when paired with nonlinear classifiers (e.g., SVM and RF), suggesting that normalization of scatter-related variability can facilitate learning of infestation-related patterns. Similar trends have been reported in wheat hyperspectral applications; for example, Caporaso [48] found SNV beneficial for improving protein prediction, likely because it normalizes scattering differences caused by kernel shape and surface structure. Nevertheless, preprocessing performance is task- and system-dependent, and the optimal choice may vary with instrument configuration, acquisition protocol, sample morphology, and modeling objectives. Consistent with this, full-band models generally improved over time (Table 1, Table 2, Table 3 and Table 4), suggesting that infestation-related spectral differences become more pronounced at later developmental stages, thereby enhancing detectability.

Based on MSC-preprocessed spectra, integrating feature selection with classification further improved efficiency and robustness. Feature selection can remove redundant variables and mitigate collinearity, which is important for high-dimensional spectral data and can reduce overfitting risk [26]. For example, Tang [49] used CARS to extract characteristic wavelengths for variety identification and reported improved performance when combined with SVM. In the present study, SPA produced a more compact wavelength subset than CARS, UVE, and IRIV, reducing computational burden; however, the SPA–SVM model showed slightly lower accuracy and Kappa values, plausibly because overly aggressive reduction may discard informative wavelengths. This observation aligns with the trade-off between parsimony and predictive performance discussed by Haghbin et al. [50]. Therefore, wavelength selection should be considered an optimization problem that balances accuracy, stability, and computational efficiency, rather than a goal of minimizing variables alone.

Across classifiers, SVM generally delivered the strongest and most stable performance, especially when coupled with MSC and appropriate feature selection. This may reflect SVM’s ability to model nonlinear class boundaries with good generalization once scatter effects and redundancy are suppressed. RF was also competitive in several settings due to robustness to noise and nonlinear relationships, though careful tuning and validation may be necessary when class imbalance exists or when models are transferred across conditions. In contrast, DT can be sensitive to training data perturbations, and KNN, being distance-based, can be affected by noise, scaling, and collinearity in high−dimensional spectral spaces; consequently, their overall performance was often inferior to SVM/RF. In practice, model choice should align with deployment priorities: compact feature DT/RF variants may be attractive when interpretability and computational efficiency are emphasized, whereas MSC + (appropriate selection) + SVM appears most promising when maximal accuracy and generalizability are required.

Several limitations should be considered for practical implementation. Reliable operation requires stable illumination, rigorous calibration, and controlled acquisition conditions, which may limit deployment in some grain storage environments. Throughput is constrained by imaging speed and kernel handling (e.g., conveying stability and orientation control), making truly high-throughput, kernel−by−kernel inspection challenging under high-flow grain streams. In addition, generalizability may be influenced by cultivar differences and fluctuations in moisture, temperature, and storage conditions, supporting the need for multi−site validation and periodic model updating or recalibration before routine use. To further improve sensitivity at early infestation stages and to strengthen mechanistic interpretation, future studies should incorporate independent physicochemical measurements as reference ground truth and explore multimodal feature fusion (e.g., spectra combined with texture/morphology) or domain adaptation strategies to enhance robustness across conditions.

## 5. Conclusions

In this study, the results demonstrated that multiplicative scatter correction (MSC) was the most effective preprocessing method, as it significantly reduced noise, enhanced spectral variability among samples, and improved overall model accuracy. In the MSC−SVM model, test set accuracies at each time point exceeded 90%, with values of 93.22%, 91.53%, 94.35%, and 94.92% for day 1, 11, 21, and 25, respectively. Notably, model performance improved as the infection progressed, suggesting that infestation features became more detectable at later stages. Further improvements were achieved by integrating feature selection algorithms with the SVM model based on MSC−preprocessed data. The MSC−CARS−SVM model achieved the highest test set accuracies for the day 1 and day 25 samples, reaching 95.48% and 96.61%, respectively. For the day 11 and day 21 samples, the MSC−IRIV−SPA−SVM model performed best, with accuracies of 94.53% and 94.92%, respectively. Compared to the full-spectrum model, the feature-filtered model demonstrated overall improvements in metrics such as test set accuracy, F1-score, and Kappa coefficient. This indicates that feature compression not only effectively enhances recognition accuracy but also strengthens the model’s stability and generalization capability. In conclusion, this study successfully distinguished between healthy and infested wheat kernels across different infestation stages, validating the feasibility and practical value of integrating hyperspectral imaging with machine learning for early, nondestructive detection of hidden pests in grain storage. Future research should expand the range of samples to include different cryptic pest species and extend validation to more complex and realistic storage scenarios, including field/warehouse conditions, to further assess the robustness and generalizability of the developed models.

## Figures and Tables

**Table 1:** DT classification performance under different preprocessing methods.

Methods	1 d				11 d				21 d				25 d			
	Train-Acc/%	Test-Acc/%	F1-Score	Kappa	Train-Acc/%	Test-Acc/%	F1-Score	Kappa	Train-Acc/%	Test-Acc/%	F1-Score	Kappa	Train-Acc/%	Test-Acc/%	F1-Score	Kappa
RAW	83.54	73.36	0.78	0.62	80.37	69.66	0.77	0.61	85.84	74.92	0.80	0.63	88.54	79.75	0.85	0.69
SG	83.65	75.84	0.80	0.63	83.12	72.92	0.79	0.63	86.92	75.62	0.83	0.67	87.65	81.88	0.87	0.72
SNV	91.77	85.31	0.87	0.71	93.45	83.62	0.85	0.68	91.53	86.94	0.89	0.73	91.77	87.70	0.90	0.77
MSC	93.46	**87.01**	**0.89**	**0.73**	93.47	**85.31**	**0.88**	**0.70**	93.28	**88.14**	**0.90**	**0.75**	94.09	**89.27**	**0.91**	**0.78**
SG-FD	93.77	80.13	0.86	0.71	86.05	81.75	0.86	0.70	93.70	83.44	0.88	0.73	94.92	85.44	0.88	0.73
SG-SD	91.04	85.31	0.87	0.71	85.32	74.05	0.82	0.66	90.80	85.57	0.89	0.73	93.43	87.57	0.89	0.75
BC	89.02	77.05	0.80	0.66	85.71	71.92	0.80	0.63	88.05	79.18	0.83	0.69	92.99	82.44	0.87	0.73
Det	93.70	83.44	0.86	0.72	92.01	76.08	0.80	0.63	91.77	84.14	0.88	0.73	93.22	85.31	0.86	0.70

Note: Bold values indicate the best results (same below).

**Table 2:** KNN classification performance under different preprocessing methods.

Methods	1 d				11 d				21 d				25 d			
	Train-Acc/%	Test-Acc/%	F1-Score	Kappa	Train-Acc/%	Test-Acc/%	F1-Score	Kappa	Train-Acc/%	Test-Acc/%	F1-Score	Kappa	Train-Acc/%	Test-Acc/%	F1-Score	Kappa
RAW	87.17	81.92	0.85	0.73	89.84	80.32	0.82	0.72	92.25	83.42	0.85	0.75	95.34	84.77	0.88	0.77
SG	94.67	82.14	0.83	0.72	90.31	81.92	0.83	0.70	91.04	85.83	0.88	0.76	94.19	86.22	0.89	0.77
SNV	92.01	85.88	0.90	0.77	93.22	84.83	0.87	0.76	92.49	87.40	0.90	0.79	95.79	89.02	0.92	0.80
MSC	95.16	**90.96**	**0.93**	**0.81**	94.67	**88.70**	**0.91**	**0.76**	94.95	**91.48**	**0.92**	**0.82**	96.82	**92.05**	**0.93**	**0.83**
SG-FD	93.7	85.96	0.90	0.76	93.88	84.09	0.86	0.75	95.64	86.26	0.90	0.77	95.40	88.75	0.92	0.80
SG-SD	95.40	83.27	0.87	0.74	92.25	83.31	0.86	0.74	92.15	87.53	0.91	0.78	93.22	87.53	0.91	0.79
BC	93.52	84.01	0.89	0.76	92.01	84.75	0.86	0.74	96.36	85.70	0.89	0.77	95.64	87.66	0.91	0.79
Det	95.61	86.53	0.90	0.79	89.35	83.27	0.85	0.73	95.16	86.07	0.90	0.77	95.44	88.07	0.91	0.79

**Table 3:** SVM classification performance under different preprocessing methods.

Methods	1 d				11 d				21 d				25 d			
	Train-Acc/%	Test-Acc/%	F1-Score	Kappa	Train-Acc/%	Test-Acc/%	F1-Score	Kappa	Train-Acc/%	Test-Acc/%	F1-Score	Kappa	Train-Acc/%	Test-Acc/%	F1-Score	Kappa
RAW	95.15	84.90	0.90	0.80	96.61	82.36	0.87	0.79	93.52	87.01	0.89	0.82	93.31	88.83	0.92	0.83
SG	94.87	85.17	0.90	0.81	95.85	83.05	0.88	0.80	94.18	88.98	0.90	0.82	94.72	89.64	0.92	0.84
SNV	96.52	91.22	0.93	0.83	94.46	86.86	0.89	0.80	93.44	90.61	0.92	0.84	96.52	90.79	0.93	0.85
MSC	97.33	**93.22**	**0.95**	**0.85**	93.34	**91.53**	**0.92**	**0.81**	98.52	**94.35**	**0.95**	**0.88**	98.72	**94.92**	**0.96**	**0.89**
SG-FD	94.87	89.64	0.92	0.82	94.97	87.44	0.91	0.81	92.18	89.34	0.94	0.85	94.36	92.09	0.94	0.87
SG-SD	95.02	87.27	0.92	0.82	95.76	86.40	0.89	0.80	95.64	90.92	0.93	0.84	93.79	91.31	0.94	0.87
BC	94.76	86.70	0.90	0.81	94.09	85.92	0.89	0.80	94.92	87.96	0.92	0.83	94.87	88.92	0.93	0.84
Det	95.46	86.35	0.90	0.81	93.13	85.66	0.90	0.82	95.34	88.22	0.93	0.84	94.03	89.18	0.93	0.84

**Table 4:** RF classification performance under different preprocessing methods.

Methods	1 d				11 d				21 d				25 d			
	Train-Acc/%	Test-Acc/%	F1-Score	Kappa	Train-Acc/%	Test-Acc/%	F1-Score	Kappa	Train-Acc/%	Test-Acc/%	F1-Score	Kappa	Train-Acc/%	Test-Acc/%	F1-Score	Kappa
RAW	93.52	83.01	0.88	0.77	94.67	81.96	0.87	0.76	93.13	85.40	0.88	0.80	93.79	86.96	0.90	0.80
SG	94.51	84.09	0.90	0.78	90.80	82.57	0.88	0.77	94.15	86.53	0.89	0.80	93.34	87.79	0.91	0.81
SNV	96.27	88.09	0.92	0.83	95.40	86.27	0.91	0.79	93.44	89.07	0.93	0.84	94.58	90.35	0.94	0.85
MSC	96.76	**92.09**	**0.93**	**0.84**	96.37	**90.40**	**0.92**	**0.80**	96.76	**92.66**	**0.94**	**0.84**	97.52	**93.22**	**0.95**	**0.86**
SG-FD	94.27	87.26	0.92	0.82	97.59	85.63	0.89	0.79	95.52	87.93	0.92	0.83	92.59	88.48	0.94	0.83
SG-SD	94.76	85.79	0.92	0.80	94.72	84.37	0.89	0.78	95.40	86.22	0.91	0.82	93.72	86.64	0.92	0.83
BC	93.79	85.96	0.91	0.80	93.61	83.83	0.87	0.76	94.43	86.53	0.91	0.81	93.52	87.92	0.91	0.82
Det	95.27	86.22	0.90	0.80	94.46	85.10	0.88	0.77	94.27	87.79	0.90	0.81	95.40	88.27	0.93	0.83

**Table 5:** Joint feature wavelength selection results.

Methods	Time/d	Number of Individuals	Bands/nm
CARS-SPA	1	10	874.3 881.1 922.1 1098.5 1345.7 1470.9 1650.1 1692.1 1695.3 1698.6
	11	13	925.6 929.0 993.6 1138.9 1219.1 1339.1 1405.2 1428.2 1643.6 1650.1 1682.4 1688.9 1692.1
	21	12	898.2 908.5 922.1 935.8 1252.7 1401.9 1461.1 1646.9 1669.5 1682.4 1685.7 1698.6
	25	17	874.3 901.7 908.5 911.9 929.0 963.0 1132.2 1222.7 1408.5 1474.2 1640.4 1656.6 1669.5 1672.7 1685.7 1688.9 1698.6
UVE-SPA	1	10	874.3 925.6 929.0 935.8 1003.8 1236.0 1349.0 1474.2 1637.2 1650.1
	11	13	922.1 925.6 929.0 935.8 993.6 1125.4 1152.3 1189.2 1411.8 1490.6 1643.6 1669.5 1679.2
	21	10	925.6 932.4 983.4 1236.0 1405.2 1470.9 1614.5 1650.1 1669.5 1698.6
	25	15	888.0 908.5 925.6 935.8 997.0 1112.0 1216.0 1339.1 1415.1 1470.9 1650.1 1663.0 1666.3 1669.5 1695.3
IRIV-SPA	1	13	935.8 942.6 969.8 1112.0 1152.3 1185.9 1216.0 1342.4 1372.2 1408.5 1461.1 1500.4 1588.5
	11	12	939.2 973.2 1108.6 1152.3 1185.9 1222.7 1329.2 1388.7 1418.4 1487.3 1611.2 1640.4
	21	11	939.2 973.2 1081.6 1142.3 1189.2 1222.7 1332.5 1395.3 1474.2 1614.5 1637.2
	25	10	939.2 969.8 1091.8 1149.0 1206.0 1342.4 1411.8 1510.2 1621.0 1643.6

**Table 6:** DT classification performance with different feature selection methods.

Methods	1 d				11 d				21 d				25 d			
	Train-Acc/%	Test-Acc/%	F1-Score	Kappa	Train-Acc/%	Test-Acc/%	F1-Score	Kappa	Train-Acc/%	Test-Acc/%	F1-Score	Kappa	Train-Acc/%	Test-Acc/%	F1-Score	Kappa
CARS	94.07	82.72	0.85	0.71	86.44	83.05	0.88	0.72	89.41	86.40	0.90	0.76	90.25	86.84	0.91	0.78
SPA	86.40	81.78	0.85	0.71	86.86	82.15	0.86	0.71	92.37	85.84	0.86	0.75	90.17	86.24	0.89	0.76
UVE	86.02	83.85	0.87	0.72	87.29	83.00	0.87	0.71	86.44	84.70	0.87	0.73	89.83	85.88	0.88	0.75
IRIV	85.84	83.47	0.86	0.71	83.90	81.59	0.86	0.70	88.89	84.99	0.88	0.75	91.53	85.53	0.88	0.74
CARS-SPA	86.86	84.42	0.87	0.74	89.44	**87.01**	**0.90**	**0.72**	90.54	**88.70**	**0.91**	**0.76**	91.41	89.18	0.91	0.78
UVE-SPA	91.53	**87.57**	**0.89**	**0.75**	86.02	83.29	0.88	0.72	89.83	85.27	0.87	0.75	93.25	**90.40**	**0.92**	**0.80**
IRIV-SPA	87.71	84.14	0.88	0.74	85.47	82.72	0.85	0.72	91.95	85.84	0.87	0.74	91.53	86.40	0.91	0.78

**Table 7:** KNN classification performance with different feature selection methods.

Methods	1 d				11 d				21 d				25 d			
	Train-Acc/%	Test-Acc/%	F1-Score	Kappa	Train-Acc/%	Test-Acc/%	F1-Score	Kappa	Train-Acc/%	Test-Acc/%	F1-Score	Kappa	Train-Acc/%	Test-Acc/%	F1-Score	Kappa Factor
CARS	90.73	88.10	0.90	0.80	84.32	82.44	0.87	0.75	94.92	86.12	0.88	0.77	96.16	89.21	0.93	0.83
SPA	96.61	88.67	0.91	0.80	87.71	**89.27**	**0.91**	**0.78**	97.03	**90.91**	**0.92**	**0.81**	95.37	**93.22**	**0.94**	**0.86**
UVE	98.73	88.67	0.92	0.82	85.78	81.30	0.86	0.72	98.73	85.27	0.88	0.76	97.03	87.72	0.92	0.79
IRIV	99.58	85.55	0.90	0.76	87.29	81.78	0.87	0.73	96.24	83.05	0.87	0.74	92.35	86.44	0.88	0.75
CARS-SPA	95.76	**91.53**	**0.93**	**0.82**	86.02	84.42	0.88	0.75	94.49	86.69	0.89	0.79	94.49	89.52	0.91	0.82
UVE-SPA	98.73	87.82	0.91	0.81	86.44	80.45	0.87	0.73	95.58	87.82	0.90	0.80	95.27	89.24	0.92	0.82
IRIV-SPA	99.15	86.12	0.90	0.79	85.17	81.02	0.85	0.71	97.46	86.97	0.90	0.80	97.88	89.45	0.93	0.83

**Table 8:** SVM classification performance with different feature selection methods.

Methods	1 d				11 d				21 d				25 d			
	Train-Acc/%	Test-Acc/%	F1-Score	Kappa	Train-Acc/%	Test-Acc/%	F1-Score	Kappa	Train-Acc/%	Test-Acc/%	F1-Score	Kappa	Train-Acc/%	Test-Acc/%	F1-Score	Kappa
CARS	97.55	**95.48**	**0.96**	**0.91**	96.47	90.48	0.93	0.85	96.19	92.78	0.96	0.89	98.83	**96.61**	**0.97**	**0.93**
SPA	95.45	91.83	0.93	0.87	94.10	90.19	0.92	0.84	96.55	92.18	0.95	0.87	95.37	93.29	0.94	0.89
UVE	96.62	94.31	0.95	0.88	95.13	91.79	0.93	0.87	95.42	93.46	0.95	0.88	96.08	94.18	0.95	0.91
IRIV	96.35	92.74	0.94	0.88	95.67	91.56	0.93	0.86	96.13	93.03	0.94	0.88	96.15	93.58	0.96	0.90
CARS-SPA	95.94	93.45	0.95	0.88	96.67	92.04	0.94	0.87	94.36	92.31	0.95	0.88	95.42	94.44	0.94	0.90
UVE-SPA	96.21	91.35	0.94	0.86	95.65	90.95	0.94	0.87	95.03	91.35	0.94	0.88	95.59	93.03	0.95	0.90
IRIV-SPA	97.32	93.19	0.94	0.88	96.06	**94.35**	**0.95**	**0.88**	97.29	**94.92**	**0.96**	**0.89**	96.78	94.44	0.95	0.91

**Table 9:** RF classification performance with different feature selection methods.

Methods	1 d				11 d				21 d				25 d			
	Train-Acc/%	Test-Acc/%	F1-Score	Kappa	Train-Acc/%	Test-Acc/%	F1-Score	Kappa	Train-Acc/%	Test-Acc/%	F1-Score	Kappa	Train-Acc/%	Test-Acc/%	F1-Score	Kappa
CARS	93.79	90.34	0.94	0.86	95.87	89.37	0.93	0.84	95.80	89.92	0.93	0.85	96.15	90.25	0.93	0.86
SPA	94.72	88.37	0.90	0.81	92.07	87.29	0.92	0.80	95.43	89.19	0.91	0.84	95.72	89.95	0.92	0.84
UVE	95.26	87.95	0.92	0.83	94.52	90.14	0.92	0.83	94.97	90.87	0.94	0.86	97.53	**94.92**	**0.96**	**0.89**
IRIV	95.43	86.25	0.91	0.80	93.36	89.71	0.92	0.83	95.51	91.39	0.93	0.85	94.37	91.55	0.92	0.86
CARS-SPA	94.18	89.61	0.91	0.81	93.79	90.37	0.91	0.82	96.72	91.37	0.91	0.84	94.90	91.92	0.92	0.84
UVE-SPA	96.58	**93.79**	**0.95**	**0.87**	95.84	**92.09**	**0.94**	**0.83**	96.16	**94.35**	**0.95**	**0.88**	95.86	92.14	0.95	0.87
IRIV-SPA	92.63	87.41	0.90	0.81	93.28	88.60	0.91	0.82	95.72	89.22	0.92	0.84	96.61	90.43	0.94	0.85

## Data Availability

The original contributions presented in this study are included in the article. Further inquiries can be directed to the corresponding author.
